# Family-based exome-wide association study of childhood acute lymphoblastic leukemia among Hispanics confirms role of *ARID5B* in susceptibility

**DOI:** 10.1371/journal.pone.0180488

**Published:** 2017-08-17

**Authors:** Natalie P. Archer, Virginia Perez-Andreu, Ulrik Stoltze, Michael E. Scheurer, Anna V. Wilkinson, Ting-Nien Lin, Maoxiang Qian, Charnise Goodings, Michael D. Swartz, Nalini Ranjit, Karen R. Rabin, Erin C. Peckham-Gregory, Sharon E. Plon, Pedro A. de Alarcon, Ryan C. Zabriskie, Federico Antillon-Klussmann, Cesar R. Najera, Jun J. Yang, Philip J. Lupo

**Affiliations:** 1 University of Texas School of Public Health, Austin Regional Campus, Austin, Texas, United States of America; 2 Department of Pharmaceutical Sciences, St. Jude Children's Research Hospital, Memphis, Tennessee, United States of America; 3 Hematologic Malignancies Program, Comprehensive Cancer Center, St. Jude Children’s Research Hospital, Memphis, Tennessee, United States of America; 4 University Hospital Copenhagen, Copenhagen, Denmark; 5 Department of Pediatrics, Section of Hematology-Oncology, Baylor College of Medicine, Houston, Texas, United States of America; 6 Michael & Susan Dell Center for Healthy Living, University of Texas Health Science Center at Houston, School of Public Health, Austin Regional Campus, Austin, Texas, United States of America; 7 Department of Biostatistics, School of Public Health, The University of Texas Health Science Center at Houston, Houston, Texas, United States of America; 8 Department of Pediatrics, University of Illinois College of Medicine at Peoria, Peoria, Illinois, United States of America; 9 Unidad Nacional de Oncología Pediátrica, Guatemala City, Guatemala; 10 School of Medicine, Francisco Marroquín University, Guatemala City, Guatemala; National Health Research Institutes, TAIWAN

## Abstract

We conducted an exome-wide association study of childhood acute lymphoblastic leukemia (ALL) among Hispanics to confirm and identify novel variants associated with disease risk in this population. We used a case-parent trio study design; unlike more commonly used case-control studies, this study design is ideal for avoiding issues with population stratification bias among this at-risk ethnic group. Using 710 individuals from 323 Guatemalan and US Hispanic families, two inherited SNPs in *ARID5B* reached genome-wide level significance: rs10821936, RR = 2.31, 95% CI = 1.70–3.14, p = 1.7×10^−8^ and rs7089424, RR = 2.22, 95% CI = 1.64–3.01, p = 5.2×10^−8^. Similar results were observed when restricting our analyses to those with the B-ALL subtype: *ARID5B* rs10821936 RR = 2.22, 95% CI = 1.63–3.02, p = 9.63×10^−8^ and *ARID5B* rs7089424 RR = 2.13, 95% CI = 1.57–2.88, p = 2.81×10^−7^. Notably, effect sizes observed for rs7089424 and rs10821936 in our study were >20% higher than those reported among non-Hispanic white populations in previous genetic association studies. Our results confirmed the role of *ARID5B* in childhood ALL susceptibility among Hispanics; however, our assessment did not reveal any strong novel inherited genetic risks for acute lymphoblastic leukemia among this ethnic group.

## Introduction

Acute lymphoblastic leukemia (ALL) is the most common malignancy among children, with B-cell ALL (B-ALL) accounting for the majority (80% to 85%) of cases [1,2]. Genome-wide association studies (GWAS) have identified several inherited genetic variants associated with childhood or adolescent ALL risk, including but not limited to single nucleotide polymorphisms (SNPs) in *ARID5B*, *IKZF1*, *CEBPE*, *CDKN2A*, *PIP4K2A*, *GATA3*, *LHPP*, and *ELK3* [3–11]. Another important risk factor for childhood ALL is Hispanic ethnicity. Children of Hispanic ethnic background have a 10% to 30% higher incidence of ALL than do non-Hispanic whites, and a rate almost two times higher than among non-Hispanic blacks [12,13]. Hispanic children with ALL also have a lower 5-year survival rate and a higher incidence of relapse than do non-Hispanic whites [14,15].

These differences in ALL incidence and outcomes among Hispanics could be due to differences in the frequency of known or novel genetic risk factors that are unique to this population. Genetic risk factors associated with Amerindian ancestry could account for increased ALL incidence and decreased ALL survival among Hispanics [15]. Furthermore, risk alleles in *ARID5B* and *GATA3* were found to be more frequent among Hispanic Americans than in European Americans [8,9], consistent with ancestry-related disparities in ALL susceptibility and treatment outcomes. However, there is much work remaining to identify the missing heritability of ALL among Hispanics.

While a majority of GWAS rely on the case-control study design, this approach is subject to population stratification bias. This bias may result in spurious associations or the masking of associations due to subgroups within a population that have different genetic profiles and/or frequencies of disease [16,17]. Population stratification bias can be particularly important among Hispanic populations, where a three-way admixture among Native American, European, and West African ancestry populations is common [18,19]. However, the family-based case-parent trio design is immune to population stratification bias, as this approach relies on evaluating disequilibrium in the transmission of alleles among affected cases and their parents, which does not vary by race and ethnicity [16,20]. Thus, the case-parent trio study design has an advantage over the case-control study when analyzing data from highly admixed populations such as Hispanics.

To determine if previous ALL GWAS findings in populations primarily of European descent are transferable to Hispanic populations, and to identify novel genetic risks for ALL for this ethnic group, we conducted an exome-wide association study (EXWAS) among Hispanics recruited in Guatemala and the Southwestern United States. We used a case-parent trio approach to attenuate the potential for population stratification bias due to admixture among this population.

## Materials and methods

### Study subjects and samples

The study population has been described previously [21]. Briefly, the population under study included Guatemalan ALL case-parent trios recruited during the period 2012–2015 from Unidad Nacional de Oncología Pediátrica (UNOP), a pediatric cancer treatment center in Guatemala City, as well as Hispanic trios recruited during the period 2003–2011 from the Texas Children’s Cancer Center (part of Texas Children’s Hospital (TCH) in Houston, Texas). Children and adolescents (ages 1 to 19 years) diagnosed with ALL and treated at either UNOP or TCH during their respective recruitment timeframes were eligible for the study. All Guatemalan ALL cases were either of indigenous Native American ancestry (Mayan; primarily K’iche’, Mam, and Q’anjob’al) or Ladino (Mestizo, with a mixed Native American and European background) ancestry. Written informed consent was obtained from parents, guardians, or patients, as appropriate. For all ALL cases, germline genomic DNA was extracted from peripheral blood samples obtained during clinical remission. Peripheral blood samples were also obtained from participating Guatemalan parents, while saliva samples were collected from parents of TCH cases. DNA extraction was performed using a Puregene DNA isolation kit by Gentra Systems.

This study was approved by the Institutional Review Boards at University of Texas Health Science Center (UTHSC), Baylor College of Medicine, and St. Jude Children’s Research Hospital, as well as by the Bioethics Committee at Facultad de Medicina, Universidad Francisco Marroquín in Guatemala.

### Genotyping and quality control (QC)

Germline genomic DNA for all samples was submitted to exome-wide genotyping using the Illumina Human Exome BeadChip (Illumina, San Diego CA). SNPs genotyped (N = 237,436) were mostly from exonic regions. Genotype calls were made using Illumina GenomeStudio Software; genotype was coded as 0, 1, or 2, indicating the number of minor alleles (an additive genetic model was assumed). Individuals with a genotype call rate <95% and case-parent trios or duos with a Mendelian error rate (i.e., Mendelian inconsistencies) >0.5% were excluded from analyses. In addition, non-autosomal SNPs, SNPs with a minor allele frequency (MAF) <1%, and SNPs with poor genotyping quality (<95% call rate) were excluded. For SNPs with a MAF of 1%-5%, a more stringent set of QC criteria determined by call rate was used (S1 Fig), and SNPs that did not meet these criteria were also excluded from analyses.

### Statistical analysis

For each SNP, associations between the inherited genotype and risk of childhood ALL were assessed with multinomial modeling, using the EMIM program [22]. This analysis compares observed case genotype distributions to expected distributions, assuming Mendelian transmission of the minor allele [20,22,23]. Multinomial modeling is mathematically equivalent to a log-linear approach [24], which has been used in several case-parent trio GWAS [25–27]. The multinomial modeling approach allows for the direct estimation of inherited effects even when one or more individuals are missing from a case-parent trio [24]. Case-parent trios, case-mother or case-father duos, parents alone, or cases alone can all be analyzed simultaneously in EMIM to estimate inherited genetic effects.

We used EMIM to estimate relative risks (RR) for each SNP, as well as corresponding 95% confidence intervals (CIs) and chi-squared values, assuming a log-additive model of inheritance. EMIM analyses stratified the data by parental mating type, inherently adjusting RR estimates for effects of population stratification [23,24]. We calculated two-tailed p-values from chi-squared values for each SNP using R, version 3.2.2. We also created Manhattan plots and Q-Q plots using R; and regional association plots using LocusZoom [28]. We used a threshold of *P*<1.0 × 10^−6^ to denote statistical significance in our analyses. The majority of ALL cases in the study cohort were of the B-ALL subtype; therefore, in addition to examining inherited variants associated with ALL in the entire study cohort, we also conducted a subgroup analysis with only B-ALL cases and families, to assess inherited genotype effects for B-ALL in particular.

In order to select cases for the TCH population, we determined genetic ancestry using STRUCTURE [15,29] on the basis of genotypes at 30,000 randomly selected SNPs, using HapMap samples (CEU, YRI, CHB/JPT) and indigenous Amerindian references [30] as ancestry populations. Hispanic Americans were defined as individuals for whom the proportion of Native American genetic ancestry was ≥10% and was also greater than the proportion of African ancestry [8].

## Results

The study cohort consists of a total of 733 individuals from 332 families: 287 families (628 individuals) from Unidad Nacional de Oncología Pediátrica (UNOP) in Guatemala, and 45 families (105 individuals) from Texas Children’s Hospital (TCH) in the United States. We excluded nine families from analysis because they did not meet Mendelian error rate criteria. The full cohort used in analyses included 323 families with 710 individuals. For 24 of these families, parental genetic data were used in the analysis, but genetic information was missing for the ALL cases themselves. Demographic and clinical characteristics of the 299 ALL cases included in this study are shown in Table 1. Of the 237,436 exonic SNPs available for analysis from the Illumina chip, 32,175 SNPs met the MAF and call rate criteria and were subsequently used in analyses (S1 File). The vast majority (99.6%) of excluded SNPs were omitted from analyses because they had a MAF of <1%.

**Table 1:** Characteristics of ALL cases used in EXWAS (N = 299).

Characteristic	All ALL cases		Guatemalan cases		US Hispanic cases	
	N	%	N	%	N	%
Race/Ethnicity						
Indigenous (Native American)	96	32.1	96	62.8	0	0.0
Ladino (Hispanic Mestizo)	162	54.2	162	37.2	0	0.0
Hispanic (Unknown type)	41	13.7	0	0.0	41	100
Sex						
Male	172	57.5	149	57.8	23	56.1
Female	127	42.5	109	42.2	18	43.9
Age						
1–10	214	71.6	177	68.6	37	90.2
>10	85	28.4	81	31.4	4	9.8
ALL subtype						
B-ALL	288	96.3	247	95.7	41	100
T-ALL	11	3.7	11	4.3	0	0.0
Total	299	100	258	100	41	100

**Fig 1:**
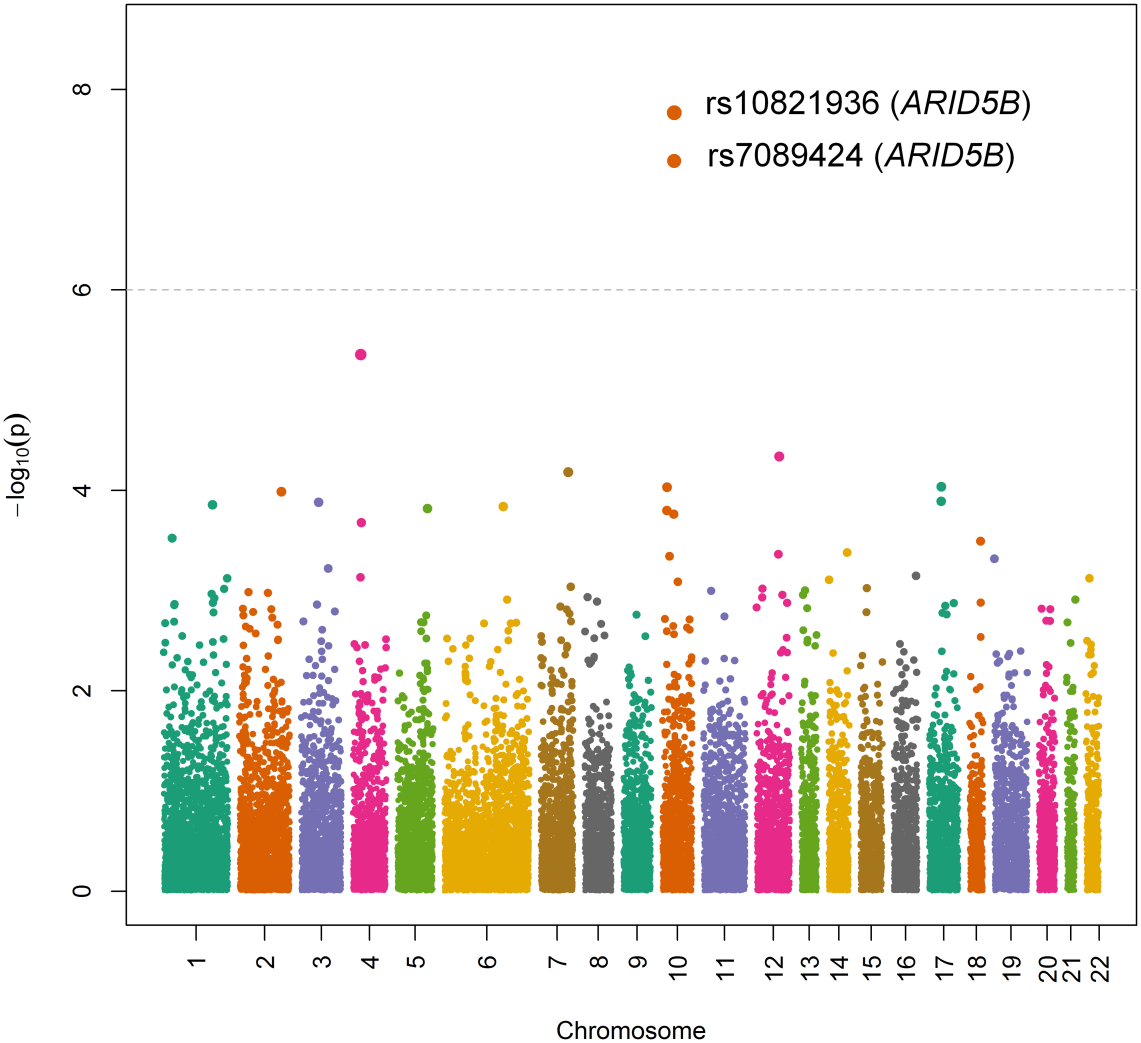
Manhattan plot of inherited genetic effects in the full study cohort.

**Fig 2:**
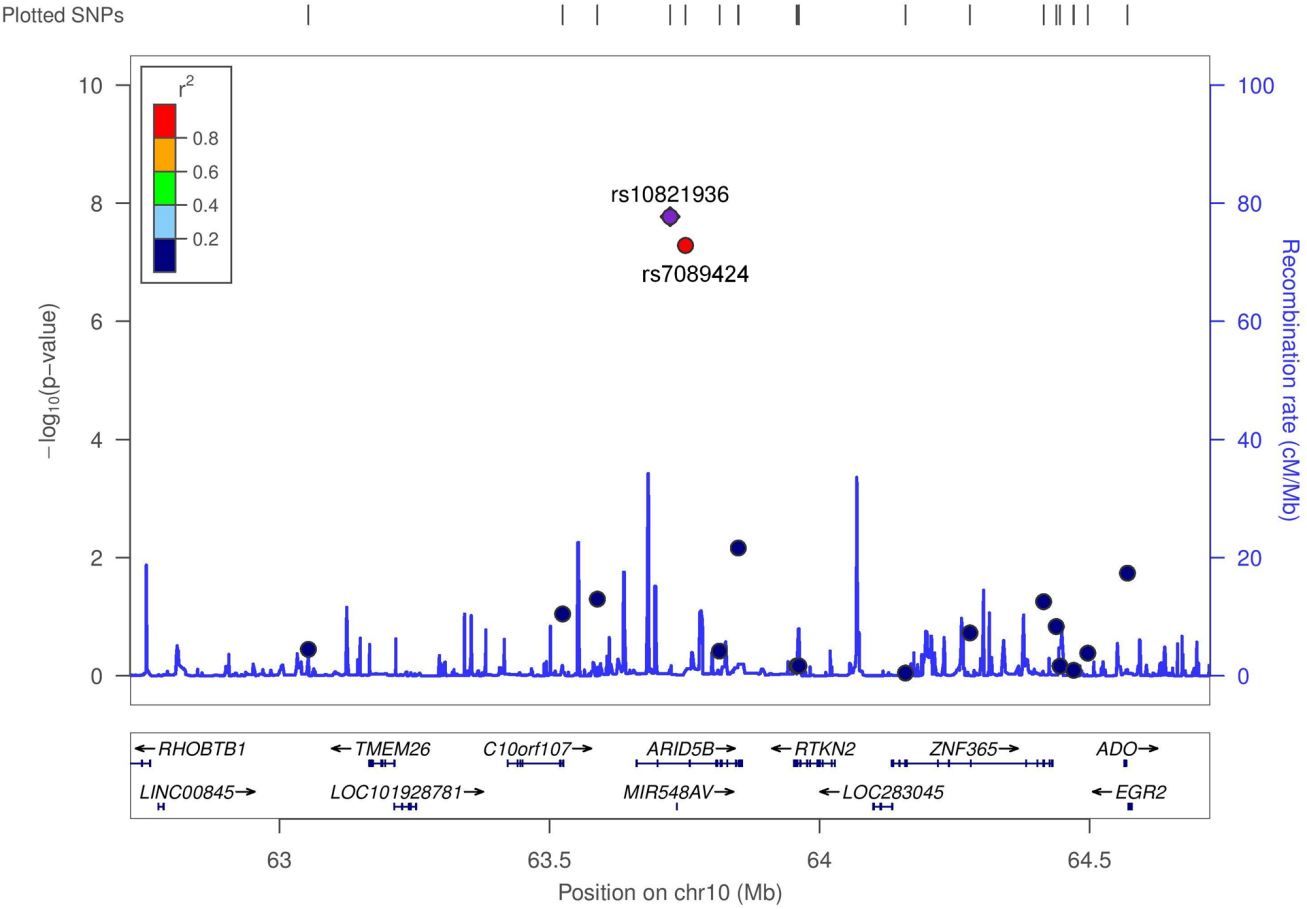
Regional association plot for *ARID5B* SNPs rs10821936 and rs7089424. The left y-axis shows the multinomial model’s association probabilities (-log10p) for the SNP of interest (rs10821936; purple diamond) as well as for nearby SNPs used in the analysis. Red shading indicates linkage disequilibrium (r^2^) between the SNP of interest and nearby markers. Recombination rates for each region, obtained from 1000 Genomes build hg19 Amerindian (AMR) data, are indicated by blue lines measured on the right y-axis. The x-axis shows the position of the SNP of interest and other markers on the chromosome, relative to the position of nearby genes.

In our exome-wide association analysis of the ALL cohort, we identified two SNPs on chromosome 10 within the *ARID5B* gene that were significantly associated with ALL risk: rs10821936 (RR = 2.31, 95% CI = 1.70–3.14, p = 1.70 × 10^−8^) and rs7089424 (RR = 2.22, 95% CI = 1.64–3.01, p = 5.19 × 10^−8^) (Fig 1 and Table 2). These two SNPs were in linkage disequilibrium (LD; r^2^ = 0.84, D’ = 0.92) in the 1000 Genomes version hg19 Amerindian (AMR) reference population [31] (Fig 2). A subcohort analysis of only B-ALL cases and families (312 families, 683 individuals) yielded very similar effect estimates for rs10821936 (RR = 2.22, 95% CI = 1.63–3.02) and rs7089424 (RR = 2.13, 95% CI = 1.57–2.88), which were also significantly associated with B-ALL susceptibility (p = 9.63 × 10^−8^ and p = 2.81 × 10^−7^, respectively) (Table 2). Results for all SNPs that were nominally significant in our analysis (p<0.05) among the entire study cohort can be found in S2 Table.

**Table 2:** Inherited effects results for *ARID5B* SNPs rs10821936 and rs7089424 (10q21.2), both for the entire ALL study cohort and for B-ALL cases and families alone.

SNP Name (Gene, Chromosome)	RAF		*P*	RR	95% CI
	Cases	Parents			
rs10821936 (*ARID5B*,10)					
Entire study cohort (n = 32 families)	0.73	0.64	1.70 x 10^−8^	2.31	(1.70, 3.14)
B-ALL subcohort (n = 312 families)	0.73	0.64	9.63 x 10^−8^	2.22	(1.63, 3.02)
rs7089424 (*ARID5B*,10)					
Entire study cohort (n = 323 families)		0.64	5.19 x 10^−8^	2.22	(1.64, 3.01)
B-ALL subcohort (n = 312 families)	0.73	0.64	2.81 x 10^−7^	2.13	(1.57, 2.88)

Because this study included Hispanic childhood ALL cases and parents from two distinct geographic locations (Guatemala and Texas), analyses were, in effect, conducted on a pooled study population. We explored results for the *ARID5B* SNPs found to be of genome-wide significance in the overall study population in both of these populations separately, and effect sizes were consistent (rs10821936: RR = 2.32 for Guatemala, RR = 2.50 for Texas; rs7089424: RR = 2.27 for Guatemala, RR = 2.35 for Texas).

## Discussion

In this family-based EXWAS of childhood ALL among those of Hispanic ancestry, we observed two inherited SNPs in *ARID5B*, rs10821936 and rs7089424, that were associated with childhood ALL. These two SNPs are in strong linkage disequilibrium with one another (r^2^ = 0.84, D’ = 0.92), most likely representing a single susceptibility locus (Fig 2). Both *ARID5B* rs7089424 and rs10821936 have been identified in previous ALL GWAS [3–5,7,8]. These two SNPs also have had some of the strongest and most statistically significant associations with ALL in GWAS analyses [4,7,8]. We reviewed whole-exome sequence data from a subset of the cases (n = 41 from TCH) described here and did not identify any recurring *ARID5B* coding variants in patients with the risk allele.

We also compared findings from our analysis to susceptibility SNPs identified in previous GWAS/EXWAS of childhood ALL (Table 3). Notably, the magnitude of the effects of rs7089424 and rs10821936 in our study were over 20% higher than estimates calculated in previous genetic association studies among primarily European (non-Hispanic white) populations.

*ARID5B* SNPs have also been evaluated among Hispanics in particular in a small subset of studies. Our results are consistent with one other study, which observed a similar effect size for rs7089424 among Hispanics with childhood B-ALL (OR = 2.33, 95%CI: 1.85–2.92) [13].

**Table 3:** Inherited effects reported in other ALL GWAS and EXWAS analyses, compared to our study results.

**SNP**	**Chr**	**Gene**	**Results—other studies**^a^			**Our study results**^b^	
			**OR (95% CI)**	***P***	**References**	**RR (95% CI)**	***P***
rs4132601	7p12.2	*IKZF1*	1.69 (1.58, 1.81)	1.2 x 10^−19^	Papaemmanuil et al. 2009	1.48 (1.09, 2.02)	0.01
			1.59 (1.34, 1.89)	1.2 x 10^−7^	Orsi et al. 2012		
			1.43 (1.30, 1.58)	8.3 x 10^−13^	Ellinghaus et al. 2012		
rs11978267	7p12.2	*IKZF1*	1.59 (1.45, 1.74)	5.3 x 10^−24^	Xu et al. 2013	1.52 (1.11, 2.07)	0.007
			(Total study pop.)				
			1.31 (1.07, 1.61)	0.01			
			(Hispanics only)				
			1.69 (1.40, 1.90)	8.8 x 10^−11^	Trevino et al. 2009		
			1.44 (1.30, 1.59)	1.1 x 10^−12^	Ellinghaus et al. 2012		
rs6964823	7p12.2	*IKZF1*	1.52 (1.41, 1.64)	6.0 x 10^−14^	Papaemmanuil et al. 2009	Not available to evaluate	
rs6944602	7p12.2	*IKZF1*	1.64 (1.37, 2.07)	3.4 x 10^−15^	Papaemmanuil et al. 2009	Not available to evaluate	
			1.42 (1.27, 1.60)	3.1 x 10^−9^	Ellinghaus et al. 2012		
rs7089424	10q21.2	*ARID5B*	1.65 (1.54, 1.76)	6.7 x 10^−19^	Papaemmanuil et al. 2009	2.22 (1.64, 3.01)	5.19 x 10^−8^
			1.42 (1.30, 1.56)	2.0 x 10^−13^	Ellinghaus et al. 2012		
			1.83 (1.55, 2.15)	6.1 x 10^−13^	Orsi et al. 2012		
rs10821936	10q21.2	*ARID5B*	1.86 (1.71, 2.03)	5.9 x 10^−46^	Xu et al. 2013	2.31 (1.70, 3.14)	1.70 x 10^−8^
			(Total study pop.)				
			1.95 (1.60, 2.38)	3.78 x 10^−11^			
			(Hispanics only)				
			1.91 (1.60, 2.20)	1.4 x 10^−15^	Trevino et al. 2009		
			1.46 (1.33, 1.60)	4.1 x 10^−15^	Ellinghaus et al. 2012		
rs7073837	10q21.2	*ARID5B*	1.58 (1.35, 1.89)	4.7 x 10^−16^	Papaemmanuil et al. 2009	Not available to evaluate	
			1.64 (1.40, 1.92)	1.0 x 10^−9^	Orsi et al. 2012		
			1.30 (1.19, 1.43)	1.5 x 10^−8^	Ellinghaus et al. 2012		
rs10740055	10q21.2	*ARID5B*	1.53 (1.41, 1.64)	5.4 x 10^−14^	Papaemmanuil et al. 2009	Not available to evaluate	
			1.75 (1.49, 2.06)	1.8 x 10^−11^	Orsi et al. 2012		
			0.76 (0.70, 0.84)	9.0 x 10^−9^	Ellinghaus et al. 2012^c^		
**SNP**	**Chr**	**Gene**	**Results—other studies**^a^			**Our study results**^b^	
			**OR (95% CI)**	***P***	**References**	**RR (95% CI)**	***P***
rs2239633	14q11.2	*CEBPE*	1.34 (1.22, 1.45)	2.9 x 10^−7^	Papaemmanuil et al. 2009	Not available to evaluate	
			0.74 (0.68, 0.82)	4.0 x 10^−10^	Ellinghaus et al. 2012^c^		
rs4982731	14q11.2	*CEBPE*	1.36 (1.24, 1.48)	9.0 x 10^−12^	Xu et al. 2013	Not available to evaluate	
			(Total study pop.)				
			1.58 (1.31, 1.91)	2.32 x 10^−6^			
			(Hispanics only)				
rs3731217	9p21.3	*CDKN2A*	0.71 (0.64, 0.78)	3.0 x 10^−11^	Sherborne et al. 2010^d^	Not available to evaluate	
rs3731249	9p21.3	*CDKN2A*	2.23 (1.90, 2.61)	9.0 x 10^−23^	Xu et al. 2015	Not available to evaluate	
rs17756311	9p21.3	*CDKN2A/B*	1.36 (1.18, 1.56)	1.4 x 10^−5^	Xu et al. 2013^e^	Not available to evaluate	
			(Total study pop.)				
			1.36 (0.94, 1.97)	0.1			
			(Hispanics only)				
rs10828317	10p12.2	*PIP4K2A*	1.23 (1.15, 1.32)	2.3 x 10^−9^	Migliorini et al. 2013	1.16 (0.75, 1.79)	0.50
rs7088318	10p12.2	*PIP4K2A*	1.40 (1.28, 1.53)	1.1 x 10^−11^	Xu et al. 2013	1.04 (0.70, 1.56)	0.83
			(Total study pop.)				
			1.42 (1.12, 1.80)	0.009			
			(Hispanics only)				
rs3824662	10p14	*GATA3*	3.85 (2.71, 5.47)	2.2 x 10^−14^	Perez-Andreu et al. 2013^f^	Not available to evaluate	
			1.31 (1.21, 1.41)	8.6 x 10^−12^	Migliorini et al. 2013		
			1.77 (1.48, 2.12)	2.8 x 10^−10^	Perez-Andreu et al. 2015		

^a^ Other study results shown are largely from GWAS conducted among individuals of European ancestry. However, Xu et al. 2013 included European-American, African-American, and Hispanic-American participants; results for this study are shown for the total study population as well as for Hispanic-Americans alone. Perez-Andreu et al. 2013 also included European-American and Hispanic participants, among others; results shown are adjusted for genetic ancestry.

^b^ Effect sizes for our entire study cohort are shown (both B-ALL and T-ALL case and parents).

^c^ These results reflect associations for the minor allele, which is not the risk allele; therefore, these results show a protective odds ratio.

^d^ OR results are for the T allele, which is not the risk allele, since results are protective. The T allele is the major (more common) allele.

^e^ Although this result has a larger p-value than the cutoff specified (1x10^-6^), it is included in the table as an additional study that has found an association between *CDKN2A* and ALL.

^f^ OR results are for a high-risk ALL subtype (Ph-like ALL), compared to non-ALL controls.

It has been observed that these *ARID5B* variants appear to be more common among Hispanics [8,13]. As would be expected, the risk allele frequencies (RAFs) observed for both rs10821936 and rs7089424 among both cases and parents in our study were higher than the risk allele frequencies reported among Hispanic and Native American reference populations (S1 Table). However, a GWAS by Xu et al. (2013) observed a RAF of 0.63 for rs10821936 among Hispanic ALL cases [8], which was closer to the RAF observed among Hispanic ALL cases for this SNP in our study (0.73). It is possible that the increased frequency of the risk allele for both rs10821936 and rs7089424 among Hispanics and Native Americans could be a factor in the increased risk of childhood ALL among these populations.

The higher magnitude of effect observed among Hispanics in this study (as compared to studies among predominantly non-Hispanic whites) could be due to a stronger effect within this population for biological reasons, which are not apparent through this study. It is also possible that the larger effect size could be due to the study design used. The case-parent trio design may be a more unbiased approach in evaluating these effects, because it produces estimates that are inherently adjusted for effects of population stratification.

*ARID5B* regulates the transcription of certain genes during embryonic development, and plays a role in the differentiation of B-lymphocyte progenitor cells [7,32]. Therefore, germline variation at this locus could alter B-lymphocyte development, thus playing a part in susceptibility to B-ALL [7,33]. Furthermore, both rs7089424 and rs10821936 may have a role in transcriptional regulation. The risk allele for rs10821936, in particular, is thought to eliminate the binding site of transcriptional factor NIT2, which could alter gene expression in *cis* [34,35].

While SNPs in *ARID5B* were the only variants to reach genome-wide level significance in our study, previously-reported SNPs *IKZF1* rs4132601 and rs11978267 were nominally significant (*P*<0.05), and their effect sizes were similar to previous assessments [3–5,7,8]. The only other SNPs previously found to be associated with ALL that were directly genotyped on the platform included *PIP4K2A* rs10828317 and rs7088318. Although *PIP4K2A* rs10828317 was not statistically significant, the effect size was comparable to that reported in Migliorini et al. (RR = 1.16 vs. 1.23) [11]. However, this was not the case for *PIP4K2A* rs7088318 (RR = 1.04 vs. 1.40) [8]. It is possible that variations in risk allele frequency among Hispanics could contribute to these differences, in part because prior assessments have largely focused on those of European ancestry. Because of the nature of the variants contained on the SNP array used in this study (i.e., the majority were less common exonic SNPs, with 86.1% of the SNPs on the chip having a MAF ≤1%), most of the SNPs associated with ALL in the literature were not available and therefore were not evaluated in this analysis.

This study should be considered in the light of certain limitations. As noted, because of the rare exonic content of the SNP chip, as well as the admixed study population, we did not perform SNP imputation. Additionally, because of the sample size, imputation of additional rare variants (and subsequent association testing) would have provided limited information. More specifically, our sample size did not allow for the detection of novel variants that are moderately associated with ALL. However, an important strength of our study was that it focused on Hispanic childhood ALL cases, in order to increase our knowledge of genetic risk factors among this higher-risk group. Populations of non-European ancestry are severely under-represented in GWAS; indeed, more than 95% of subjects in previous GWAS have been of European descent [19,36]. Because Hispanic children carry the burden of both a higher incidence of childhood ALL and worse outcomes of the disease than non-Hispanic whites or blacks [37,38], it is especially important to conduct exome- and genome-wide assessments for this disease among this particular ethnic group. Furthermore, a case-parent trio study design was used; unlike the case-control design employed by many GWAS, this approach is not subject to population stratification bias and is thus more likely to yield valid conclusions when assessing inherited genetic effects among Hispanic or other genetically diverse populations [16,17].

In summary, this is the first family-based study to evaluate inherited genetic variations associated with childhood ALL among those with Hispanic ancestry. These results confirm that *ARID5B* plays an important role in childhood ALL susceptibility in this high-risk population, and that the risk of childhood ALL associated with *ARID5B* rs10821936 and rs7089424 variants may be greater for Hispanics than among European populations. The study’s focus on Hispanic populations addresses an important gap in information about childhood ALL genetic risk factors for Hispanics.

## Supporting information

S1 Table Risk allele frequencies (RAFs) of *ARID5B* rs10821936 and rs7089424 for each of the groups used in the analysis, as well as for selected reference populations. (DOCX)

S2 Table Results for the 100 most highly significant SNPs in the inherited effects analysis, entire ALL study cohort. (DOCX)

S1 Fig QC criteria based upon a combination of MAF and call rate (for those SNPs with a MAF <5%). (TIF)

S1 File Compressed file folder containing allele frequencies for genotyped subjects and raw statistical results for SNPs included in analysis. (ZIP)
