# Supplementary material for: Family-based exome-wide association study of childhood acute lymphoblastic leukemia among Hispanics confirms role of ARID5B in susceptibility
Source: PLoS One. 2017 Aug 17;12(8):e0180488. doi: 10.1371/journal.pone.0180488 (PMC5560704; doi:10.1371/journal.pone.0180488)
Supplement: S1 Table — (DOCX) [file pone.0180488.s001.docx]

**S1 Table. Risk allele frequencies (RAFs) of *ARID5B* rs10821936 and rs7089424 for each of the groups used in the analysis, as well as for selected reference populations.**

| Group | | RAF | | |
| --- | --- | --- | --- | --- |
|  |  | rs10821936 (risk allele = C) |  | rs7089424 (risk allele = G) |
| Entire study cohort | |  |  |  |
|  | Cases | 0.73 |  | 0.73 |
|  | Parents | 0.64 |  | 0.64 |
| B-ALL subcohort | |  |  |  |
|  | Cases | 0.73 |  | 0.73 |
|  | Parents | 0.64 |  | 0.64 |
| 1000 Genomes Phase 3 AMR | | 0.48 |  | 0.48 |
| HapMap CEU | | 0.31 |  | 0.30 |
| HapMap CHB | | 0.28 |  | 0.28 |
| HapMap JPT | | 0.32 |  | 0.33 |
| HapMap MEX | | 0.37 |  | 0.38 |
| HapMap YRI | | 0.21 |  | 0.23 |
