# Supplementary material for: Family-based exome-wide association study of childhood acute lymphoblastic leukemia among Hispanics confirms role of ARID5B in susceptibility
Source: PLoS One. 2017 Aug 17;12(8):e0180488. doi: 10.1371/journal.pone.0180488 (PMC5560704; doi:10.1371/journal.pone.0180488)
Supplement: S2 Table — (DOCX) [file pone.0180488.s002.docx]

S2 Table. Results for the 100 most highly significant SNPs in the inherited effects analysis (entire ALL study cohort).

| SNP name | Chr | Gene | MAF | | RR | 95% CI | *P* |
| --- | --- | --- | --- | --- | --- | --- | --- |
|  |  |  | Cases | Parents |  |  |  |
| rs10821936 | 10 | ARID5B^a^ | 0.73 | 0.64 | 2.31 | (1.70, 3.14) | 1.70×10^-8^ |
| rs7089424 | 10 | ARID5B^a^ | 0.73 | 0.64 | 2.22 | (1.64, 3.01) | 5.19×10^-8^ |
| rs10018622 | 4 |  | 0.24 | 0.35 | 0.51 | (0.38, 0.69) | 4.43×10^-6^ |
| rs10777332 | 12 |  | 0.26 | 0.23 | 2.00 | (1.41, 2.83) | 4.61×10^-5^ |
| rs3735007 | 7 | ZC3HAV1 | 0.19 | 0.26 | 0.53 | (0.39, 0.73) | 6.59×10^-5^ |
| rs4430796 | 17 | HNF1B | 0.32 | 0.37 | 0.57 | (0.43, 0.76) | 9.21×10^-5^ |
| rs838759 | 10 | EBLN1 | 0.23 | 0.29 | 0.57 | (0.43, 0.76) | 9.32×10^-5^ |
| rs13420690 | 2 | SPAG16 | 0.34 | 0.40 | 0.60 | (0.46, 0.78) | 1.03×10^-4^ |
| rs7501939 | 17 | HNF1B | 0.31 | 0.33 | 0.57 | (0.42, 0.76) | 1.29×10^-4^ |
| rs2166801 | 3 | ROBO2 | 0.15 | 0.19 | 2.05 | (1.39, 3.03) | 1.32×10^-4^ |
| rs12128544 | 1 | KCNT2 | 0.00 | 0.03 | 0.20 | (0.08, 0.50) | 1.39×10^-4^ |
| N/A | 6 | KIAA0240 | 0.05 | 0.04 | 0.22 | (0.09, 0.53) | 1.45×10^-4^ |
| rs4704970 | 5 |  | 0.00 | 0.05 | 0.31 | (0.16, 0.58) | 1.52×10^-4^ |
| rs2243897 | 10 | EBLN1 | 0.24 | 0.31 | 0.59 | (0.44, 0.78) | 1.60×10^-4^ |
| rs915188 | 10 |  | 0.15 | 0.25 | 0.53 | (0.37, 0.74) | 1.73×10^-4^ |
| rs78136490 | 4 | GRXCR1 | 0.02 | 0.05 | 0.31 | (0.16, 0.59) | 2.10×10^-4^ |
| rs2229493 | 1 | HSPG2 | 0.00 | 0.01 | 13408.58 | (1.84×10^-30^, 9.78×10^-37^) | 3.01×10^-4^ |
| rs2228349 | 1 | HSPG2 | 0.00 | 0.01 | 13408.58 | (1.84×10^-30^, 9.78×10^-37^) | 3.01×10^-4^ |
| rs3180227 | 18 | SERPINB3 | 0.11 | 0.22 | 0.55 | (0.39, 0.76) | 3.22×10^-4^ |
| rs8006467 | 14 |  | 0.03 | 0.07 | 0.35 | (0.20, 0.64) | 4.17×10^-4^ |
| rs12424429 | 12 | SLC6A15 | 0.19 | 0.09 | 0.44 | (0.27, 0.70) | 4.33×10^-4^ |
| N/A | 10 | NRP1 | 0.08 | 0.05 | 3.04 | (1.51, 6.14) | 4.53×10^-4^ |
| rs3745925 | 19 | MADCAM1 | 0.03 | 0.19 | 0.55 | (0.39, 0.77) | 4.82×10^-4^ |
| N/A | 3 |  | 0.48 | 0.49 | 1.58 | (1.21, 2.06) | 6.00×10^-4^ |
| rs10863168 | 16 |  | 0.47 | 0.47 | 1.61 | (1.21, 2.13) | 7.13×10^-4^ |
| rs2711941 | 4 | KLHL5 | 0.16 | 0.22 | 0.59 | (0.43, 0.80) | 7.39×10^-4^ |
| rs688034 | 22 | SEZ6L | 0.08 | 0.09 | 2.52 | (1.41, 4.52) | 7.55×10^-4^ |
| rs2642993 | 1 | ZNF670-ZNF695 | 0.00 | 0.01 | 0.07 | (0.01, 0.57) | 7.57×10^-4^ |
| rs1760898 | 14 | TEP1 | 0.45 | 0.33 | 1.62 | (1.21, 2.15) | 7.81×10^-4^ |
| rs11001819 | 10 | C10orf11 | 0.08 | 0.12 | 0.49 | (0.32, 0.75) | 8.20×10^-4^ |
| rs77510079 | 7 | SSPO | 0.02 | 0.04 | 0.27 | (0.12, 0.61) | 9.19×10^-4^ |
| rs2271437 | 15 | SLC28A2 | 0.31 | 0.24 | 1.71 | (1.23, 2.38) | 9.46×10^-4^ |
| N/A | 12 |  | 0.00 | 0.04 | 6.47 | (1.53, 27.26) | 9.58×10^-4^ |
| rs2793086 | 1 | TSNAX-DISC1 | 0.03 | 0.05 | 0.36 | (0.19, 0.67) | 9.60×10^-4^ |
| rs636437 | 13 |  | 0.34 | 0.35 | 0.61 | (0.46, 0.83) | 9.93×10^-4^ |
| rs10839752 | 11 | SYT9 | 0.45 | 0.39 | 1.52 | (1.18, 1.97) | 0.001 |
| rs719593 | 2 |  | 0.10 | 0.03 | 3.52 | (1.49, 8.32) | 0.001 |
| rs6711382 | 2 | NEB | 0.11 | 0.09 | 2.20 | (1.33, 3.64) | 0.001 |
| rs4593773 | 1 |  | 0.44 | 0.39 | 1.61 | (1.20, 2.17) | 0.001 |
| rs62619824 | 13 | MTMR6 | 0.11 | 0.11 | 2.06 | (1.30, 3.27) | 0.001 |
|  |  |  |  |  |  |  |  |
| S2 Table. Results for the 100 most highly significant SNPs in the inherited effects analysis,  entire ALL study cohort (continued). | | | | | | | |
| SNP name | Chr | Gene | MAF | | RR | 95% CI | *P* |
|  |  |  | Cases | Parents |  |  |  |
| rs61743416 | 12 | RFX4 | 0.03 | 0.04 | 0.33 | (0.16, 0.66) | 0.001 |
| rs4831837 | 8 |  | 0.23 | 0.35 | 0.63 | (0.47, 0.83) | 0.001 |
| rs7957558 | 12 | PLBD1 | 0.00 | 0.02 | 10.43 | (1.39, 78.24) | 0.001 |
| rs2820289 | 1 | NAV1 | 0.29 | 0.16 | 1.93 | (1.27, 2.93) | 0.001 |
| rs239798 | 6 | FAM83B | 0.05 | 0.08 | 0.45 | (0.27, 0.73) | 0.001 |
| rs9475077 | 6 | FAM83B | 0.05 | 0.08 | 0.45 | (0.27, 0.73) | 0.001 |
| rs10854485 | 21 | PRMT2 | 0.03 | 0.10 | 0.47 | (0.30, 0.75) | 0.001 |
| rs1060242 | 8 | ADHFE1 | 0.18 | 0.31 | 0.59 | (0.43, 0.82) | 0.001 |
| rs1020694 | 18 | SERPINB13 | 0.11 | 0.22 | 0.59 | (0.42, 0.82) | 0.001 |
| rs1169076 | 12 | WDR66 | 0.40 | 0.44 | 1.58 | (1.19, 2.11) | 0.001 |
| rs16841081 | 1 | ASPM | 0.00 | 0.01 | 0.08 | (0.01, 0.62) | 0.001 |
| rs4789659 | 17 |  | 0.24 | 0.19 | 1.75 | (1.23, 2.49) | 0.001 |
| N/A | 1 | AIM1L | 0.19 | 0.19 | 1.78 | (1.23, 2.57) | 0.001 |
| rs264111 | 3 | MAGI1 | 0.23 | 0.23 | 1.69 | (1.21, 2.36) | 0.001 |
| rs3795686 | 1 | CEP85 | 0.19 | 0.18 | 1.76 | (1.23, 2.54) | 0.001 |
| rs1071682 | 17 | HIGD1B,EFTUD2 | 0.05 | 0.09 | 0.47 | (0.29, 0.76) | 0.001 |
| rs2906645 | 7 | GATS,PVRIG | 0.16 | 0.30 | 0.58 | (0.42, 0.82) | 0.001 |
| N/A | 12 | KDM5A | 0.05 | 0.04 | 3.18 | (1.42, 7.09) | 0.001 |
| rs9534264 | 13 | ZC3H13 | 0.15 | 0.22 | 0.59 | (0.43, 0.82) | 0.002 |
| rs3732149 | 2 | HS1BP3 | 0.05 | 0.11 | 0.51 | (0.33, 0.77) | 0.002 |
| rs1135711 | 20 | TMX4 | 0.00 | 0.01 | 0.08 | (0.01, 0.62) | 0.002 |
| N/A | 2 |  | 0.21 | 0.28 | 0.61 | (0.44, 0.83) | 0.002 |
| rs6032474 | 20 |  | 0.10 | 0.18 | 0.57 | (0.40, 0.81) | 0.002 |
| rs4731702 | 7 |  | 0.42 | 0.34 | 1.61 | (1.19, 2.18) | 0.002 |
| rs3853735 | 3 | EGFEM1P | 0.32 | 0.40 | 0.64 | (0.48, 0.85) | 0.002 |
| N/A | 2 |  | 0.02 | 0.02 | 0.20 | (0.06, 0.60) | 0.002 |
| rs753414 | 15 | SHF | 0.18 | 0.27 | 0.59 | (0.43, 0.83) | 0.002 |
| rs3850625 | 1 | CACNA1S | 0.08 | 0.07 | 2.37 | (1.33, 4.25) | 0.002 |
| rs13695 | 17 | TOP2A | 0.05 | 0.06 | 0.38 | (0.21, 0.71) | 0.002 |
| rs13438232 | 7 | CLCN1 | 0.15 | 0.22 | 0.58 | (0.42, 0.82) | 0.002 |
| N/A | 17 | EFCAB13 | 0.18 | 0.11 | 1.95 | (1.26, 3.02) | 0.002 |
| rs56108623 | 9 | ERCC6L2 | 0.21 | 0.21 | 1.81 | (1.23, 2.67) | 0.002 |
| rs1117324 | 2 |  | 0.13 | 0.16 | 1.87 | (1.24, 2.83) | 0.002 |
| rs2053028 | 5 | FAT2 | 0.03 | 0.08 | 0.43 | (0.25, 0.75) | 0.002 |
| rs499037 | 11 | OR10V1 | 0.21 | 0.20 | 1.74 | (1.21, 2.49) | 0.002 |
| N/A | 2 | ITGA6 | 0.45 | 0.41 | 1.56 | (1.17, 2.08) | 0.002 |
| rs2274110 | 10 | MCM10 | 0.03 | 0.08 | 0.44 | (0.26, 0.75) | 0.002 |
| rs869809 | 10 |  | 0.23 | 0.33 | 0.62 | (0.46, 0.84) | 0.002 |
| rs2071056 | 20 | E2F1 | 0.42 | 0.46 | 1.55 | (1.17, 2.05) | 0.002 |
| rs6017667 | 20 | SPINT4 | 0.10 | 0.18 | 0.57 | (0.40, 0.82) | 0.002 |
| N/A | 3 | CAND2 | 0.19 | 0.09 | 2.21 | (1.29, 3.78) | 0.002 |
|  |  |  |  |  |  |  |  |
| **S2 Table. Results for the 100 most highly significant SNPs in the inherited effects analysis,**  **entire ALL study cohort (continued).** | | | | | | | |
| SNP name | Chr | Gene | MAF | | RR | 95% CI | *P* |
|  |  |  | Cases | Parents |  |  |  |
| rs709060 | 7 | SSPO | 0.02 | 0.04 | 0.30 | (0.14, 0.67) | 0.002 |
| N/A | 1 | AIM1L | 0.19 | 0.20 | 1.73 | (1.21, 2.50) | 0.002 |
| rs40986 | 5 | FBXL21 | 0.19 | 0.18 | 0.60 | (0.43, 0.83) | 0.002 |
| rs2824790 | 21 | TMPRSS15 | 0.21 | 0.23 | 1.71 | (1.20, 2.43) | 0.002 |
| rs12515587 | 5 | PCDH1 | 0.03 | 0.07 | 0.43 | (0.25, 0.75) | 0.002 |
| rs13190932 | 6 | TRAF3IP2-AS1 | 0.02 | 0.05 | 0.37 | (0.19, 0.71) | 0.002 |
| exm6998 | 1 | MEGF6 | 0.23 | 0.36 | 1.55 | (1.16, 2.07) | 0.002 |
| rs2821008 | 6 |  | 0.00 | 0.02 | 9.66 | (1.28, 72.90) | 0.002 |
| rs204890 | 6 | ATF6B | 0.00 | 0.01 | 9.75 | (1.29, 73.78) | 0.002 |
| rs1269851 | 6 | ATF6B | 0.00 | 0.01 | 9.75 | (1.29, 73.78) | 0.002 |
| exm2264301 | 8 |  | 0.26 | 0.31 | 0.63 | (0.46, 0.85) | 0.002 |
| exm2269194 | 2 |  | 0.29 | 0.26 | 1.64 | (1.18, 2.27) | 0.002 |
| exm825888 | 10 | PCDH15 | 0.00 | 0.02 | 0.17 | (0.05, 0.62) | 0.002 |
| rs6737027 | 2 | SPDYA | 0.06 | 0.15 | 0.53 | (0.36, 0.80) | 0.002 |
| exm856771 | 10 | NRAP | 0.34 | 0.24 | 1.62 | (1.18, 2.23) | 0.002 |
| exm191568 | 2 | LOC388946 | 0.02 | 0.01 | 444.73 | (1.55×10^-5^, 1.3×10^10^) | 0.002 |
| exm336286 | 3 | MORC1 | 0.08 | 0.11 | 2.01 | (1.24, 3.23) | 0.002 |
| exm862966 | 10 | LHPP | 0.13 | 0.16 | 0.59 | (0.42, 0.83) | 0.002 |
| rs12430350 | 13 |  | 0.03 | 0.19 | 0.55 | (0.37, 0.82) | 0.002 |

^a^Risk allele frequencies are shown for rs10821936 and rs7089424, rather than MAFs (risk alleles for these SNPs are the major allele in the study population).
