# Supplementary figures and images for: Family-based exome-wide association study of childhood acute lymphoblastic leukemia among Hispanics confirms role of ARID5B in susceptibility
Source: PLoS One. 2017 Aug 17;12(8):e0180488. doi: 10.1371/journal.pone.0180488 (PMC5560704; doi:10.1371/journal.pone.0180488)

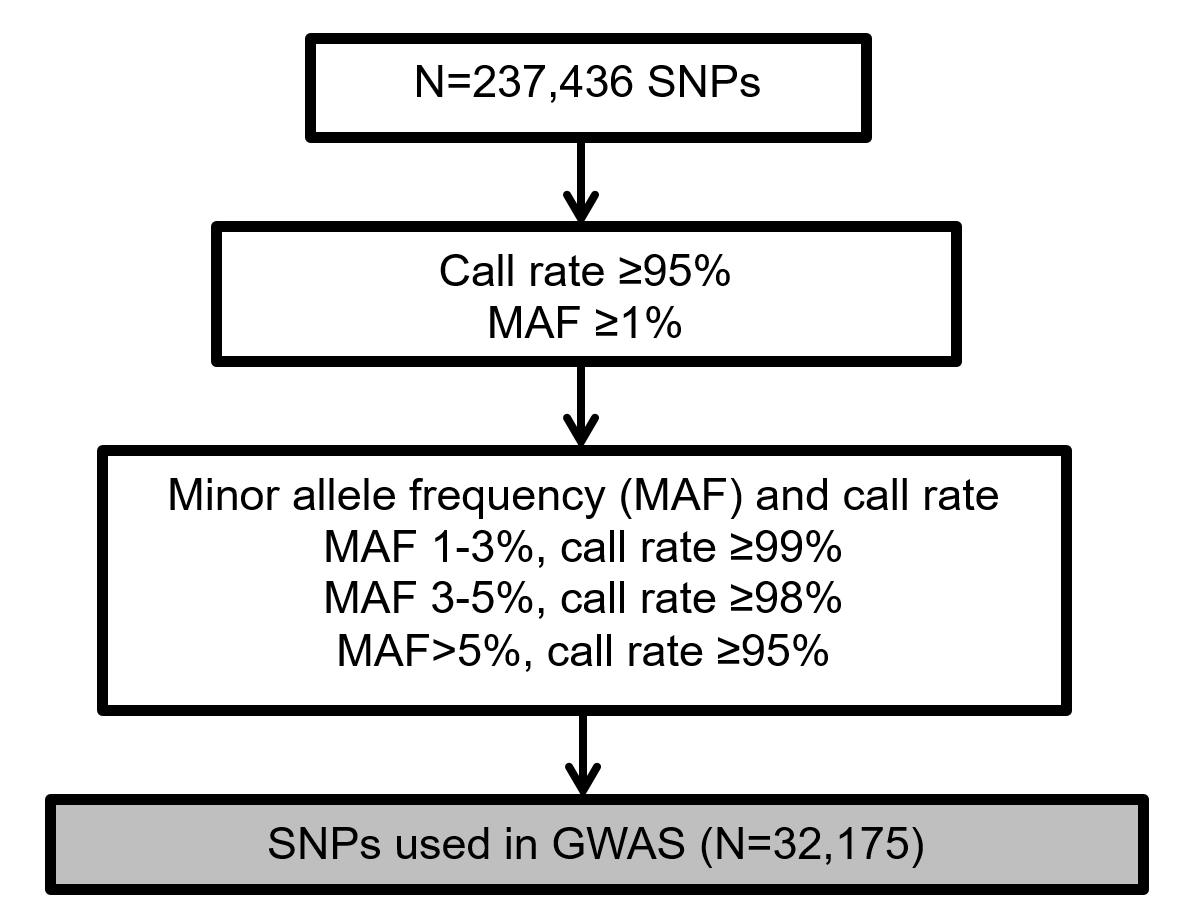

Supplement: S1 Fig — (TIF) [file pone.0180488.s003.tif]
